# Virulence determinant and antimicrobial resistance traits of Emerging MDR Shiga toxigenic *E.coli* in diarrheic dogs

**DOI:** 10.1186/s13568-022-01371-4

**Published:** 2022-03-17

**Authors:** Abdelazeem M. Algammal, Reham M. El-Tarabili, Khyreyah J. Alfifi, Amenah S. Al-Otaibi, Marwa E. Abo Hashem, Mamdouh M. El-Maghraby, Ahmed E. Mahmoud

**Affiliations:** 1 Department of Bacteriology, Immunology and Mycology, Faculty of Veterinary Medicine, Suez Canal University, Ismailia, 41522 Egypt; 2 Biology Department, Faculty of Science, Tabuk University, Tabuk, 71491 Saudi Arabia; 3 Department of Animal Medicine, Faculty of Veterinary Medicine, Suez Canal University, Ismailia, 41522 Egypt

**Keywords:** STEC, Dogs, Antibiogram, MDR, Antimicrobial resistance genes, Virulence determinants

## Abstract

Shiga-toxigenic *Escherichia* *coli* (STEC) is incriminated in severe hemorrhagic enteritis in dogs, which is considered a veterinary and public health alarm. To investigate the prevalence, antimicrobial resistance patterns, virulence determinants, and distribution of antimicrobial resistance genes in STEC strains isolated from dogs: 80 fecal samples were obtained from diseased dogs suffering from hemorrhagic diarrhea from pet animal clinics in Ismailia governorate, Egypt. The obtained samples were examined bacteriologically. Moreover, the retrieved isolates were tested for serogrouping, Congo-red binding, antimicrobial resistance, and PCR-based determination of virulence and antimicrobial resistance genes. The prevalence of *E.* *coli* in the examined diseased dogs was 23.75% (19/80). The serogrouping of the recovered isolates revealed that 84.2% of the tested isolates were distributed into three serogroups: O146 (36.8%), O111 (31.5%), and O26 (15.7%). Meanwhile, three isolates were untypable (15.8%). Moreover, all the tested *E.* *coli* serovars were positive for CR-binding. PCR revealed that the prevalence of *stx*1, *eae*A, *hly*A, and *stx*2 virulence genes was 100%, 100%, 100%, and 47.3%, respectively. Our findings revealed that 31.5% of the recovered isolates showed MDR to five antimicrobial classes and harbored *bla*_TEM_, *bla*_CTX-M_, *tet*A, *tet*B, and *sul*1 genes. Alarmingly, three isolates were carbapenem-resistant. Two strains harbored the *bla*_KPC_ gene, while one strain carried the *bla*_NDM-1_ gene. Concisely, as far as we know, this is the first study that reported the existence of MDR-STEC in dogs in Egypt. The *stx*1 gene is the most predominant Shiga toxin gene that accompanied the STEC isolated from hemorrhagic enteritis in dogs. The emerging MDR-STEC in dogs commonly harbors *bla*_TEM_, *bla*_CTX-M_, *sul*1, *tet*A, *tet*B, and *qnr*A resistance genes. Meropenem, levofloxacin, and tigecycline exhibited talented antimicrobial activity against MDR-STEC isolated from dogs.

## Introduction

Acute diarrhea in canine puppies is one of the most common and serious gastrointestinal problems, potentially leading to severe dehydration and death (Duijvestijn et al. 2016). *Escherichia* *coli* (*E.* *coli*) is found frequently in the gastrointestinal tract of both humans and animals as natural commensals (Cho et al. 2006). Based on the mechanism of disease occurrence, the pathogenic *E.* *coli* is classified into Enterohemorrhagic *E.* *coli* (EHEC), Enterotoxigenic *E.* *coli* (ETEC), Enteropathogenic *E.* *coli* (EPEC), Enteroaggregative *E.* *coli* (EAEC), and Enteroinvasive *E.* *coli* (Kaper et al. 2004). Moreover, regarding the virulence determinants, *E.* *coli* are divided into three main categories: 1-Commensals *E.* *coli*, 2-Intraintestinal-pathogenic *E.* *coli*, and 3-Extraintestinal-pathogenic *E.* *coli* (ExPEC). Each pathotype has specific virulence factors that play a major role in developing the disease, such as the production of toxins, hemolysins, siderophores, proteases, and adhesions (Pitout 2012).

Shiga toxigenic *E.* *coli* (STEC) is a common zoonotic diarrheagenic pathotype that can produce Shiga-toxins family (Stxs) cytotoxins (Melton-Celsa 2014). STEC possesses over 400 serotypes, some of which are associated with severe illnesses in animals and humans (Torres et al. 2018). The most predominant STEC serovars are O157:H7 and non-O157 serogroups (Such as O45, O121, O111, O26, O145, and O103) (Miko et al. 2014; Conrad et al. 2014). The emergence of STEC in dogs was reported with a prevalence ranging from 4 to 15.5% (Bentancor et al. 2007; Galarce 2019). The serotype O157: H7 is incriminated in most food-borne outbreaks in humans. In contrast, the non-O157 serovars are commonly associated with severe illness in animals (Rangel et al. 2005; Hedican et al. 2009).

The most important virulent determinants associated with STEC include 1-Shiga-toxins (stx1 and stx2) encoded by Shiga-toxins genes *stx*1 and *stx*2, respectively, 2-Intimin encoded by *E.* *coli* attaching and effacing gene (*eae*), and 3-Hemolysin that is necessary for STEC pathogenicity, especially *hly*A (Puño-Salvadori et al. 2003; Puño-Sarmiento et al. 2013). The polymerase chain reaction is an accurate, sensitive, and consistent diagnostic assay used for bacterial identification and the determination of virulence determinants and antimicrobial resistance genes (Carvalho et al. 2021).

The multidrug resistance (MDR) patterns have increased worldwide, especially in the last decade. Several recent epidemiological studies have revealed the emergence of MDR bacterial pathogens from different origins that reflect a public health threat (Vega-Manriquez et al. 2020; Dazio et al. 2021). Multidrug resistance is common in *E.* *coli* and is primarily caused by the presence of antimicrobial resistance genes such as; *bla*_TEM_ (responsible for penicillin-resistance), *bla*_CTX-M_ (cephalosporins-resistance), *tet*A and *tet*B (tetracycline-resistance), *qnr*A, *qnr*B, and *qnr*S (Plasmid-mediated quinolone resistance), and *sul*1 (sulfonamide resistance). Although the emergence of carbapenem-resistant bacterial pathogens is low, it has increased recently worldwide. The carbapenem-resistance is mediated mainly by *bla*_KPC_ and *bla*_NDM-1_ genes (Wedley et al. 2017; Peterhans et al. 2018; Alba et al. 2021).

The present study aimed to elucidate the clinical findings in naturally infected puppies with STEC. Also, to detect the rate, Cong-red binding, antimicrobial resistance patterns, virulence and antimicrobial resistance genes in STEC isolated from the examined puppies.

## Materials and methods

### Clinical examination and sampling

The clinical examination was conducted in small animal clinics in Ismailia province, Egypt, from March 2020 to June 2020. A total of 80 puppies (*n* = 80; 45 male and 35 female German Shepherd dogs) aged between 2 and 3 months old with an average weight of 5–10 kg were involved in the present study. The examined puppies suffered from bloody diarrhea. The clinical examination was performed according to the procedures described previously (Cote and Cohn 2019). Eighty fecal swabs were collected from the examined dogs under aseptic conditions into tryptic soy broth (Oxoid, UK), then transported to the microbiology laboratory as soon as possible for further bacteriological identification.

### Isolation and identification of *E. coli*

The collected fecal swabs were inoculated in MacConkey broth (Difco, USA) and left incubated at 37 °C for 24 h. A loopful of the incubated broth was streaked on MacConkey agar and Eosin Methylene Blue agar (EMB) plates (Difco, USA) and left incubated at 37 °C for 24 h. The purified colonies were identified according to their cultural characteristics, Grams's staining, hemolysis on blood agar, motility test, and biochemical tests (lactose fermentation, oxidase, Voges-Proskauer, indole, methyl-red, citrate-utilization, catalase, urease, and H_2_S production) as previously described by MacFaddin (MacFaddin 1985).

### Serogrouping of the retrieved isolates

The serogrouping of the recovered isolates was carried out at the Serology Division of the AHRI, Giza, Egypt, using the slide agglutination test for detection of O antigen, where polyvalent and specific monovalent *E.* *coli* antisera were involved (Mast Group Ltd.-Co., Merseyside, UK) (Starr 1986).

### Congo-red binding test

To evaluate the in-vitro invasiveness and virulence of the retrieved isolates, the Congo-red binding assay was performed using tryptic soy agar (containing 0.03% Congo-red dye) (Difco, USA). The retrieved isolates were streaked on tryptic soy agar and left incubated at 37 °C for 24 h. The positive result is indicated by the appearance of red colonies, as previously reported by Panigrahy and Yushen (1990).

### Antimicrobial susceptibility testing of *E. coli*

The in-vitro antimicrobial resistance patterns of the recovered isolates were determined on Mueller–Hinton agar (Oxoid, UK) using the disc diffusion method according to the procedures of (CLSI 2017). *E.* *coli*-ATCC 35218 was involved as a quality control strain. A blank disc impregnated with 30 μl of sterile distilled water was used as a negative control. The main concentration of bacteria was 1 × 10^8^ CFU/mL, which is equivalent to the McFarland 0.5 Turbidity Standard. Moreover, twelve antimicrobial discs were tested include ceftazidime (CAZ) (30 μg), amikacin (AK) (30 μg), cefotaxime (CTX) (30 μg), tigecycline (TCG) (15 μg), trimethoprim-sulfamethoxazole (SXT) (25 μg), amoxicillin-clavulanic acid (AMC) (30 μg), tetracycline (TE) (30 μg), levofloxacin (LEV) (5 μg), aztreonam (ATM) (30 μg), amoxicillin (AMX) (30 μg), colistin sulfate (CT) (10 μg), and meropenem (MEM) (10 μg) (ThermoFisher Scientific, USA). The tested antimicrobial agents are the most commonly used antibiotics in Egypt in both the veterinary and health sectors. The examined isolates are categorized as MDR (MDR: resistant to ≥ one antibiotic in ≥ three antimicrobial classes) as previously described by Magiorakos et al. (2012).

### Screening of virulence and antimicrobial resistance genes using PCR

The bacterial DNA was extracted consistently with the manufacturer’s guidelines of the GenElute DNA Kit (Sigma Aldrich, USA). Briefly, the DNA Kit uses spin column technology. An optimized combination of mechanical and chemical lysis is used to disrupt bacterial cells subsequently, and carefully designed buffer chemistry promotes the binding of the released DNA to the silica membrane. Finally, the bacterial DNA is eluted from the column after washing away unwanted components. Nanodrop (Nanodrop 1000, Thermo Scientific, UK) was used to quantify genomic DNA templates, which were then adjusted to 100 ng l^−1^ and stored at − 20 °C until used for PCR. The PCR reactions were carried out in a volume of 25 μl (12.5 μl of Taq Green Master Mix 2X (Promega, Wisconsin, USA), one μl of each primer, 5 μl of DNA template, and PCR grade water up to 25 μL. Positive controls were involved in all reactions (*E.* *coli* strains previously isolated and kindly supported by the Animal Health Research Institute, Egypt). Besides, a reaction with no DNA template was used as a negative control. The oligonucleotide sequences (ThermoFisher Scientific, USA), specific amplicon size, and cycling conditions are shown in Table 1. Amplified PCR products were screened by 1.5% (w/v) agarose gel electrophoresis (Applichem GmbH, Darmstadt, Germany) for 45 min at 100 V in 1X TAE buffer (0.04 M Tris, 0.0001 M EDTA, pH 8.0), visualized using 15 µL of ethidium bromide (Sigma Aldrich, USA) and photographed under a UV transilluminator.

**Table 1:** Oligonucleotides sequences and cycling conditions of PCR assay

Target genes	Primers sequences	Amplicon size (bp)	Reaction volume (µL)	Amplification (35 cycles)			References
				Denat	Annealing	Extension	
*eae*A (Intimin)	ATGCTTAGTGCTGGTTTAGG GCCTTCATCATTTCGCTTTC	248	25	94 ˚C 30 s	51 ˚C 30 s	72 ˚C 30 s	(Bisi-Johnson et al. 2011)
*hly*A (Hemolysin)	AACAAGGATAAGCACTGTTCTGGCT ACCATATAAGCGGTCATTCCCGTCA	1177	25	94 ˚C 30 s	54 ˚C 40 s	72 ˚C 45 s	(Piva et al. 2003)
*stx*1 (Shiga-toxin 1)	ACACTGGATGATCTCAGTGG CTGAATCCCCCTCCATTATG	614	50	94 ˚C 30 s	58 ˚C 40 s	72 ˚C 45 s	(Dipineto et al. 2006)
*stx*2 (Shiga-toxin 2)	CCATGACAACGGACAGCAGTT CCTGTCAACTGAGCAGCACTTTG	779					
*sul*1 (Sulfonamides resistance)	CGGCGTGGGCTACCTGAACG GCCGATCGCGTGAAGTTCCG	433	25	94 ˚C 30 s	55 ˚C 45 s	72 ˚C 45 s	(Ibekwe et al. 2011)
*tet*A (Tetracycline resistance)	GGTTCACTCGAACGACGTCA CTGTCCGACAAGTTGCATGA	576	50	94 ˚C 30 s	55 ˚C 40 s	72 ˚C 45 s	(Randall 2004)
*tet*B (Tetracycline resistance)	CCTCAGCTTCTCAACGCGTG GCACCTTGCTCATGACTCTT	634					
*qnr*A (Quinolones resistance)	AGAGGATTTCTCACGCCAGG TGCCAGGCACAGATCTTGAC	580	50	95 ˚C 1 min	54 ˚C 1 min	72 ˚C 1 min	(Cattoir et al. 2007)
*qnr*B (Quinolones resistance)	GGMATHGAAATTCGCCACTG TTTGCYGYYCGCCAGTCGAA	264					
*qnr*S (Quinolones resistance)	GCAAGTTCATTGAACAGGGT TCTAAACCGTCGAGTTCGGCG	428					
*bla*_CTX-M_ (Cephalosporins resistance)	ATGTGCAGYACCAGTAARGTKATGGC TGGGTRAARTARGTSACCAGAAYCAGCGG	593	25	94 ˚C 30 s	54 ˚C 40 s	72 ˚C 45 s	(Archambault et al. 2006)
*bla*_TEM_ (Penicillin resistance)	ATCAGCAATAAACCAGC CCCCGAAGAACGTTTTC	516	25	94 ˚C 30 s	54 ˚C 40 s	72 ˚C 45 s	(Colom et al. 2003)
*bla*_KPC_ (Carbpenem resistance)	ATGTCACTGTATCGCCGTCT TTACTGCCCGTTGACGCCC	892	25	94 ˚C 1 min	55 ˚C 1 min	72 ˚C 1 min	(Xia et al. 2012)
*bla*_NDM-1_ (Carbpenem resistance)	GGCGGAATGGCTCATCACGA CGCAACACAGCCTGACTTTC	287	25	94 ˚C 30 s	55 ˚C 30 s	72 ˚C 30 s	

### Statistical analyses

The Chi-square was carried out to analyze the resistance phenotype using the statistical analysis software (SAS, software version 9.4), and significance was determined at a *p* value < 0.05. The results of antimicrobial susceptibility testing were graphed by a heatmap through GraphPad Software (version 8.0.1, GraphPad Software Inc., La Jolla, CA, USA). A heatmap with hierarchical clustering was performed to visualize the overall distribution of virulence genes among recovered isolates using the “heatmap” package in R software (version 4.0.2; https://www.r-project.org/).

The association among various variables (resistance phenotypes and antimicrobial resistance genes) was carried out using Spearman correlations. The correlation coefficients and their *p*-values were visualized using a correlation plot.

The correlation analyses and visualization were done using the R packages rcorr: (https://hbiostat.org/R/Hmisc/), corrplot: (https://github.com/taiyun/corrplot/), and ggcorrplot: (https://github.com/taiyun/ggcorrplot/).

## Results

### Clinical manifestations of hemorrhagic diarrhea in the examined dogs

The prominent recorded clinical signs included anorexia, lethargy, depression, vomiting, foul-smelling bloody watery diarrhea, and severe dehydration manifested by retardation of skin turgor tests for up to 10 s. Besides, the affected dogs showed an elevation in body temperature (≥ 39.5 °C) that gradually decreased with the progression of the disease.

### The phenotypic characteristics and the prevalence of *E. coli* in the examined dogs

Morphologically, the recovered isolates were moderate-sized Gram-negative, motile, and non-spore-forming bacilli. On MacCkoney agar, the obtained isolates were lactose fermenters (pink colonies). Moreover, the colonies were metallic sheen colonies on EMB and were hemolytic on Blood agar. Besides, all the retrieved isolates were negative for oxidase, H_2_S production, citrate-utilization, urease production, and Voges-Proskauer tests. Concurrently, they were positive for lactose fermentation, methyl-red, catalase, indole, and nitrate reduction tests. The prevalence of *E.* *coli* in the examined fecal swabs collected from diseased dogs was 23.75% (19/80).

### *E. coli* serogrouping

The serogrouping of the recovered isolates revealed that 16 isolates (16/19, 84.2%) belonged to three O serogroups: O146 (7/19, 36.8%), O111 (6/19, 31.5%), and O26 (3/19, 15.8%). Furthermore, three isolates (3/19, 15.8%) were untypable. Statistically, there was no significant difference (*p* ˃ 0.05) in the frequency of the recovered *E.* *coli* serovars.

### Congo-red binding assay

Our findings revealed that 84.2% of the tested isolates (16/19) were positive for Congo-red binding. All the examined *E.* *coli* serotypes were positive: O111 (6/19), O146 (7/19), and O26 (3/19). Moreover, the untyped isolates were negative for CR-binding (3/19).

### Antimicrobial resistance profiles of the recovered *E. coli*

**Fig. 1:**
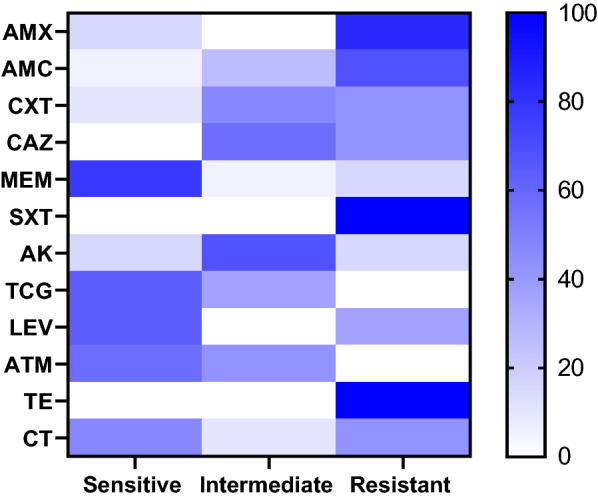
The heat map describes the susceptibility of the recovered *E.* *coli* isolates to various tested antimicrobial agents

The antimicrobial susceptibility proved that the tested isolates showed remarkable resistance to trimethoprim-sulfamethoxazole, tetracycline, amoxicillin, amoxicillin-clavulanic acid, ceftazidime, cefotaxime, and colistin sulfate with a prevalence of 100%, 100%, 84.2%, 68.4%, 42.1%, 42.1%, and 42.1%, respectively. Moreover, the recovered isolates exhibited intermediate resistance to amikacin (68.4%). Besides, three isolates (15.8%) were resistant to meropenem (carbapenem-resistant). Our findings revealed that the retrieved isolates were sensitive to meropenem (78.94%), levofloxacin, and tigecycline (63.2% for each), as illustrated in Table 2 and Fig. 1. The statistical analysis revealed a significant difference in the susceptibility of the recovered strains to different involved antimicrobial agents (*p* < 0.001).

**Table 2:** Antibiogram profiles of the recovered *E.* *coli* isolates (*n* = 19)

Antimicrobial classes	Antimicrobial agents	Sensitive		Intermediate		Resistant	
		*n*	%	*n*	%	*n*	%
Penicillins	Amoxicillin (AMX)	3	15.8	0	0	16	84.2
	Amoxicillin-Clavulanic acid (AMC)	1	5.2	5	26.3	13	68.4
Cephalosporins	Cefotaxime (CTX)	2	10.5	9	47.3	8	42.1
	Ceftazidime (CAZ)	0	0	11	57.9	8	42.1
Carbapenems	Meropenem (MEM)	15	78.94	1	5.26	3	15.8
Sulfonamide	Trimethoprim-Sulfamethoxazole (SXT)	0	0	0	0	19	100
Aminoglycoside	Amikacin (AK)	3	15.8	13	68.4	3	15.7
Glycylcycline	Tigecycline (TCG)	12	63.2	7	36.8	0	0
Fluroquinolones	Levofloxacin (LEV)	12	63.2	0	0	7	36.8
Monobactam	Aztreonam (ATM)	11	57.9	8	42.1	0	0
Tetracycline	Tetracycline (TE)	0	0	0	0	19	100
Polymyxins	Colistin sulfate (CT)	9	47.3	2	10.5	8	42.1
*p* value		*p* < 0.0001*		*p* < 0.0001*		*p* < 0.0001*	
Chi square		62.235		54.143		58.231	

*Significant differences were noticed between AMX and TCG: X^2^ = 7.6842, *p* = 0.02145, AMX and ATM: X^2^ = 7.6842, *p* = 0.02145, AMX and SXT: X^2^ = 7.6842, *p* = 0.02145, AMX and TE: X^2^ = 7.6842, *p* = 0.02145, AMX and LEV: X^2^ = 6.107, *p* = 0.047, CTX and CAZ: X^2^ = 15.119, *p* < 0.0001, CAZ and CT: X^2^ = 15.119, *p* < 0.0001, MEM and TCG: X^2^ = 8.8947, *p* = 0.00286, MEM and ATM: X^2^ = 8.8947, *p* = 0.00286, MEM and TE: X^2^ = 8.8947, *p* = 0.00286, MEM and SXT: X^2^ = 8.8947, *p* = 0.00286, MEM and AK: X^2^ = 12.223, *p* < 0.0001, SXT and AK: X^2^ = 8.8947, *p* = 0.00286, TE and AK: X^2^ = 8.8947, *p* = 0.00286, AK and ATM: X^2^ = 8.8947, *p* = 0.00286, AK and TCG: X^2^ = 8.8947, *p* = 0.00286

**Fig. 2:**
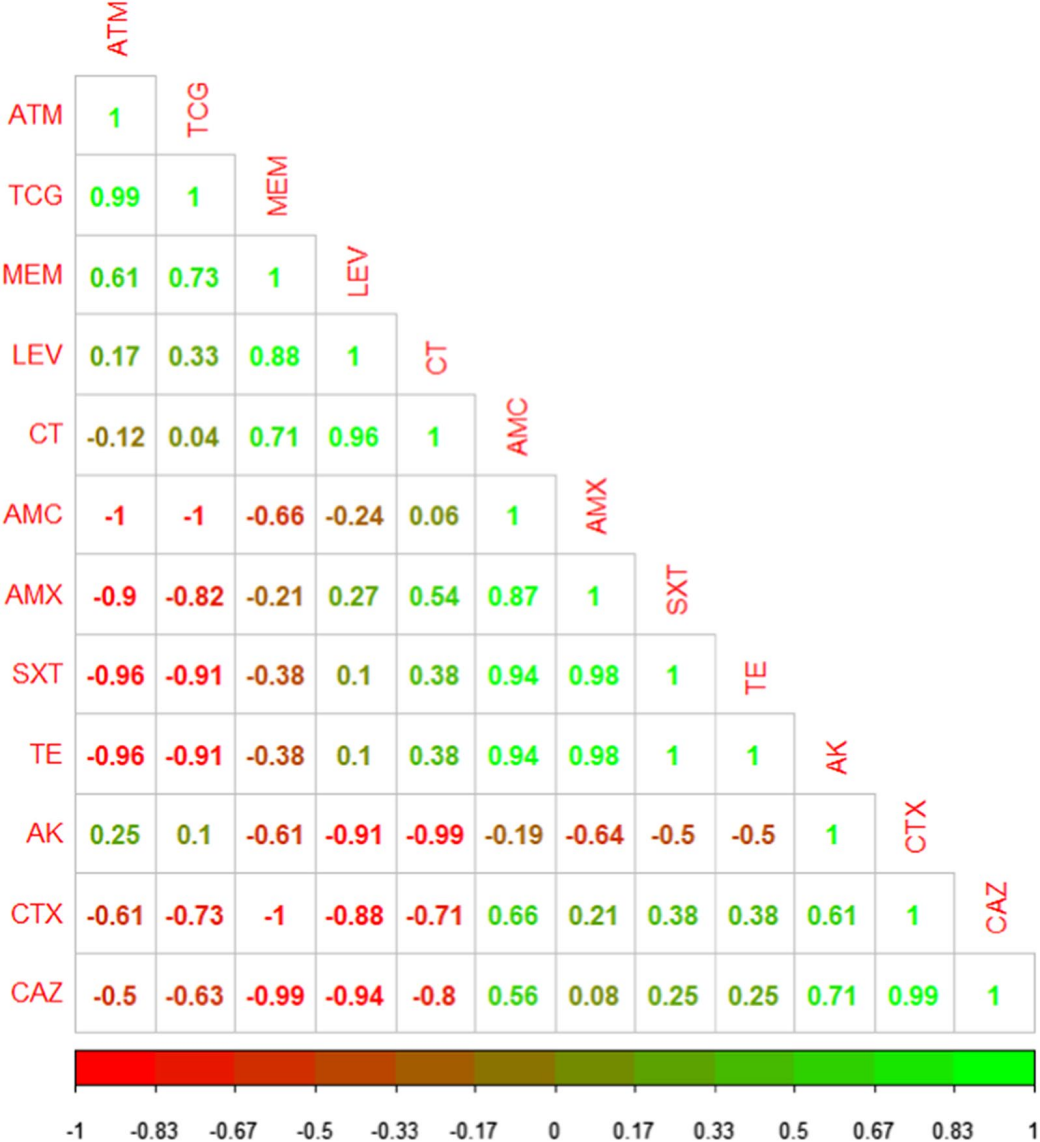
The heat map describes the correlation among different antimicrobial agents

The correlation coefficient was determined among various tested antimicrobial agents. As illustrated in Fig. 2**,** significant positive correlations were observed between: TCG and ATM (r = 0.99); CAZ and CTX (r = 0.99); AMX, TE, and SXT (r = 0.98); LEV and CT (r = 0.96); AMC, TE, and SXT (r = 0.94); LEV and MEM (r = 0.88); AMX and AMC (r = 0.87).

### Screening of virulence and antimicrobial resistance genes in the retrieved isolates (*n* = 19)

**Fig. 3:**
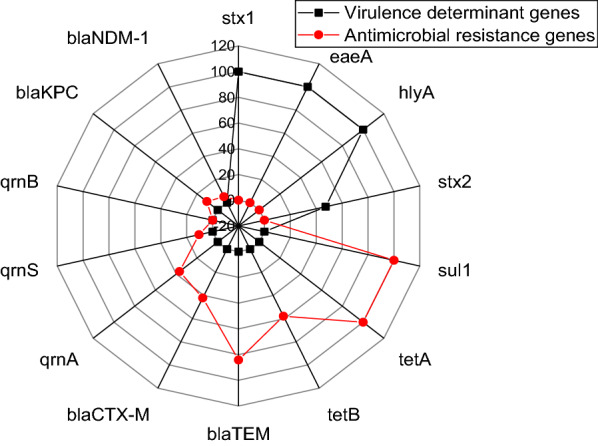
The dissemination of virulence determinants and antimicrobial resistance genes between the retrieved *E.* *coli* isolates

Using PCR, the recovered isolates possess the virulence genes; *stx*1, *eae*A, *hly*A, and *stx*2, with a prevalence of 100%, 100%, 100%, and 47.3%, respectively. Concerning the antimicrobial resistance genes, the recovered isolates possess *bla*_TEM_, *bla*_CTX-M_, *tet*A, *tet*B, *sul*1, *qnr*A, and *qnr*S resistance genes with a prevalence of 84.2%, 42.1%, 100%, 57.9%, 100%, 36.8%. 10.5%, respectively. Our findings revealed that *tet*A gene was detected in all tested *E.* *coli* strains either found alone or in combination with *tet*B gene (11/19, 57.9%). Furthermore, *qnr*A gene was detected in seven tested strains alone or combined with *qnr*S gene (2/19. 10.5%). Besides, *qnr*B gene was absent in all tested *E.* *coli* strains. Concerning carbapenem resistance genes, the *bla*_KPC_ gene was found in two isolates (2/19, 10.5%), while *bla*_NDM-1_ (1/19, 5.26%) was found only in one isolate (Fig. 3).

### Phenotypic multidrug-resistance profiles and the frequency of the antimicrobial resistance genes among the recovered isolates

In the present study, 84.2% (16/19) of the recovered isolates were recognized as multidrug-resistant (MDR: resistant to ≥ one antimicrobial agent in ≥ three different classes). Our findings revealed that 31.5% (6/19) of the recovered isolates showed MDR to 5 antimicrobial classes (sulfonamides: trimethoprim-sulfamethoxazole, tetracyclines: tetracycline, penicillins: amoxicillin and amoxicillin-clavulanic acid, cephalosporins: ceftazidime and cefotaxime, and polymyxins: colistin sulfate) and harbored *bla*_TEM_, *bla*_CTX.-M_, *tet*A, *tet*B, and *sul* genes.

Furthermore, 26.3% (5/19) of the recovered isolates expressed MDR to 4 different classes (tetracyclines: tetracycline, sulfonamides: trimethoprim-sulfamethoxazole, penicillins: amoxicillin and quinolones: levofloxacin) and harbored *bla*_TEM_, *tet*A, *tet*B, *qnr*A, and *sul*1 genes.

Besides, 10.5% (2/19) of the retrieved isolates displayed MDR to 7 different classes (tetracyclines: tetracycline, sulfonamides: trimethoprim-sulfamethoxazole, penicillins: amoxicillin carbapenems: meropenem, aminoglycoside: amikacin, cephalosporins: ceftazidime and cefotaxime, and polymyxins: colistin sulfate) and harbored *bla*_TEM_, *bla*_CTX-M_, *bla*_KPC_, *tet*A, and *sul*1 genes.

Moreover, 10.5% (2/19) of the recovered isolates expressed MDR to 4 different classes (tetracyclines: tetracycline, sulfonamides: trimethoprim-sulfamethoxazole, penicillins: amoxicillin and quinolones: levofloxacin) and harbored *bla*_TEM_, *tet*A, *qnr*A, *qnr*S, and *sul*1 genes. Furthermore, one strain displayed MDR to 5 antimicrobial classes (tetracyclines: tetracycline, sulfonamides: trimethoprim-sulfamethoxazole, penicillins: amoxicillin carbapenems: meropenem, and aminoglycosides: amikacin) and harbored *bla*_TEM_, *bla*_NDM-1_, *tet*A, and *sul*1 genes as illustrated in Table 3. Our findings revealed that three recovered *E.* *coli* strains were carbapenem-resistant. Two strains harbored the *bla*_KPC_ gene, and one strain carried the *bla*_NDM-1_ gene.

**Table 3:** The distribution of multidrug-resistance patterns and the corresponding-resistance genes among the tested *E.* *coli* isolates (*n* = 19)

No. of strains	%	Resistance type	Phenotypic multidrug resistance patterns	Antimicrobial-resistance genes
6	31.5	MDR	Five classes AMX and AMC CAZ and CTX SXT TE CT	*bla*_TEM_, *bla*_CTX.-M_, *tet*A, *tet*B, and *sul*1
5	26.3	MDR	Four classes AMX and AMC LEV SXT TE	*bla*_TEM_, *tet*A, *tet*B,, *qnr*A, and *sul*1
2	10.5	MDR	Seven classes AMX and AMC CAZ and CTX MEM AK SXT TE CT	*bla*_TEM_, *bla*_CTX-M_, *bla*_KPC_, *tet*A, and *sul*1
2	10.5	MDR	Four classes AMX LEV SXT TE	*bla*_TEM_, *tet*A, *qnr*A, *qnr*S, and *sul*1
1	5.2	MDR	Five classes AMX MEM AK SXT TE	*bla*_TEM_, *bla*_NDM-1_, *tet*A, and *sul*1

**Fig. 4:**
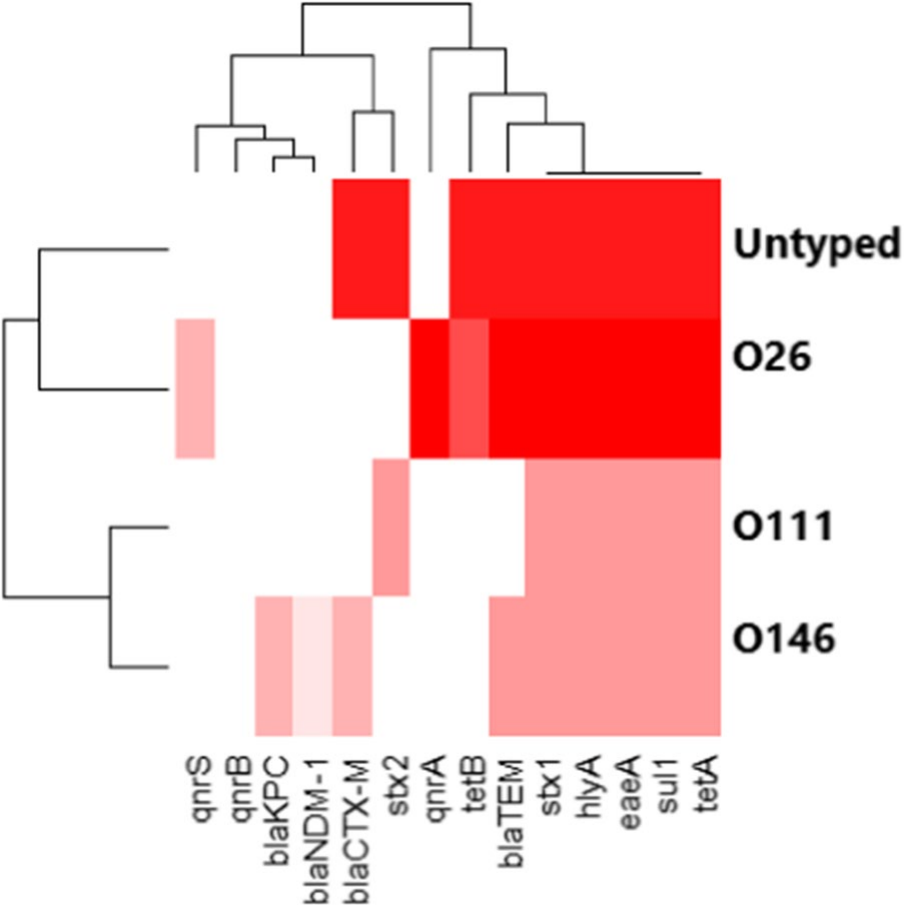
The heat map shows the correlation between virulence genes, the antimicrobial-resistance genes, and the retrieved STEC serovars

The distribution of phenotypic resistance patterns, virulence, and antimicrobial resistance genes between the recovered *E.coli* serotypes (*n* = 19) is illustrated at the serovar level in Table 4 and Fig. 4.

**Table 4:** The distribution of Phenotypic resistance patterns, virulence and antimicrobial resistance genes between the retrieved *E.* *coli* serogroups (*n* = 19)

Serogroups	Phenotypic resistance	Genotypic resistance	MAR index	Virulence genes	Resistance type
O111	SXT, AMX, AMC, TE, CAZ, CTX, CT	*bla*_CTX-M_, *bla*_TEM_, *sul*1, *tet*B, *tet*A	0.58	*stx*1, *stx*2 *eae*A *hly*A	MDR
O146	SXT, AMX, AMC, LEV, TE	*bla*_TEM_, *sul*1, *tet*A, *tet*B, *qnr*A	0.41	*eae*A, *hly*A, *stx*1	MDR
Untyped	SXT, TE	*sul*1, *tet*A	0.2	*stx*2 *eae*A, *hly*A, *stx*1	R
O111	SXT, AMX, AMC, CTX, CAZ, TE, CT	*bla*_CTX-M_, *bla*_TEM_, *tet*A, *tet*B, *sul*1	0.58	*stx*1, *stx*2 *eae*A, *hly*A	MDR
Untyped	SXT, TE	*sul*1, *tet*A	0.2	*stx*1, *stx*2 *eae*A, *hly*A	R
O111	SXT, AMX, AMC, CTX, CAZ, TE, CT	*bla*_CTX-M_, *bla*_TEM_, *tet*A, *tet*B, *sul*1	0.58	*stx*1, *stx*2 *eae*A, *hly*A,	MDR
O111	SXT, AMX, AMC, CTX, CAZ, TE, CT	*bla*_CTX-M_,,*bla*_TEM_, *sul*1, *tet*B, *tet*A	0.58	*stx*1, *stx*2 *eae*A, *hly*A	MDR
O146	SXT, AMX, AMC, LEV, TE	*bla*_TEM_, *sul*1, *tet*A, *tet*B, *qnr*A	0.41	*eae*A, *hly*A, *stx*1	MDR
O146	SXT, AMX, LEV, TE	*bla*_TEM_, *sul*1, *tet*A, *qnr*S,*qnr*A	0.41	*eae*A, *hly*A, *stx*1	MDR
O146	SXT, AMX, AMC, LEV, TE	*bla*_TEM_, *sul*1, *tet*A, *tet*B, *qnr*A	0.41	*eae*A, *hly*A, *stx*1	MDR
O146	SXT, AMX, AMC, LEV, TE	*bla*_TEM_, *sul*1, *tet*A, *tet*B, *qnr*A	0.41	*eae*A, *hly*A, *stx*1	MDR
O26	SXT, AMX, AMC, CAZ, CTX, MEM, CT, AK, TE	*bla*_CTX-M_, *bla*_TEM_, *sul*1, *bla*_KPC_, *tet*A	0.75	*eae*A, *hly*A, *stx*1	MDR
O146	SXT, AMX, AMC, LEV, TE	*bla*_TEM_, *sul*1, *tet*A, *tet*B, *qnr*A	0.41	*eae*A,*hly*A, *stx*1	MDR
O111	SXT, AMX, AMC, CAZ, CTX, TE	*bla*_CTX-M_, *bla*_TEM_, *sul*1, *tet*B, *tet*A	0.58	*stx*1, *stx*2, *eae*A, *hly*A	MDR
O26	SXT, AMX, MEM, AK, TE	*bla*_TEM_, *sul*1, *bla*_NDM-1_, *tet*A	0.5	*eae*A,*hly*A, *stx*1	MDR
O146	SXT, AMX, LEV, TE	*bla*_TEM_, *sul*1, *tet*A, *qnr*S, *qnr*A	0.41	*eae*A, *hly*A, *stx*1	MDR
O26	SXT, AMX, AMC, CAZ, CTX, MEM, CT, AK, TE	*bla*_CTX-M_,*bla*_TEM_, *sul*1, *bla*_KPC_, *tet*A	0.75	*eae*A, *hly*A, *stx*1	MDR
O111	SXT, AMX, AMC, CTX, CAZ, TE, CT	*bla*_CTX-M_, *bla*_TEM_, *sul*1, *tet*B, *tet*A	0.6	*stx*1, *stx*2 *eae*A, *hly*A	MDR
Untyped	SXT, TE	*sul*1, *tet*A	0.2	*eae*A, *stx*2, *hly*A, *stx*1	R

*MAR* multiple antibiotic resistance = (a/b)

**Fig. 5:**
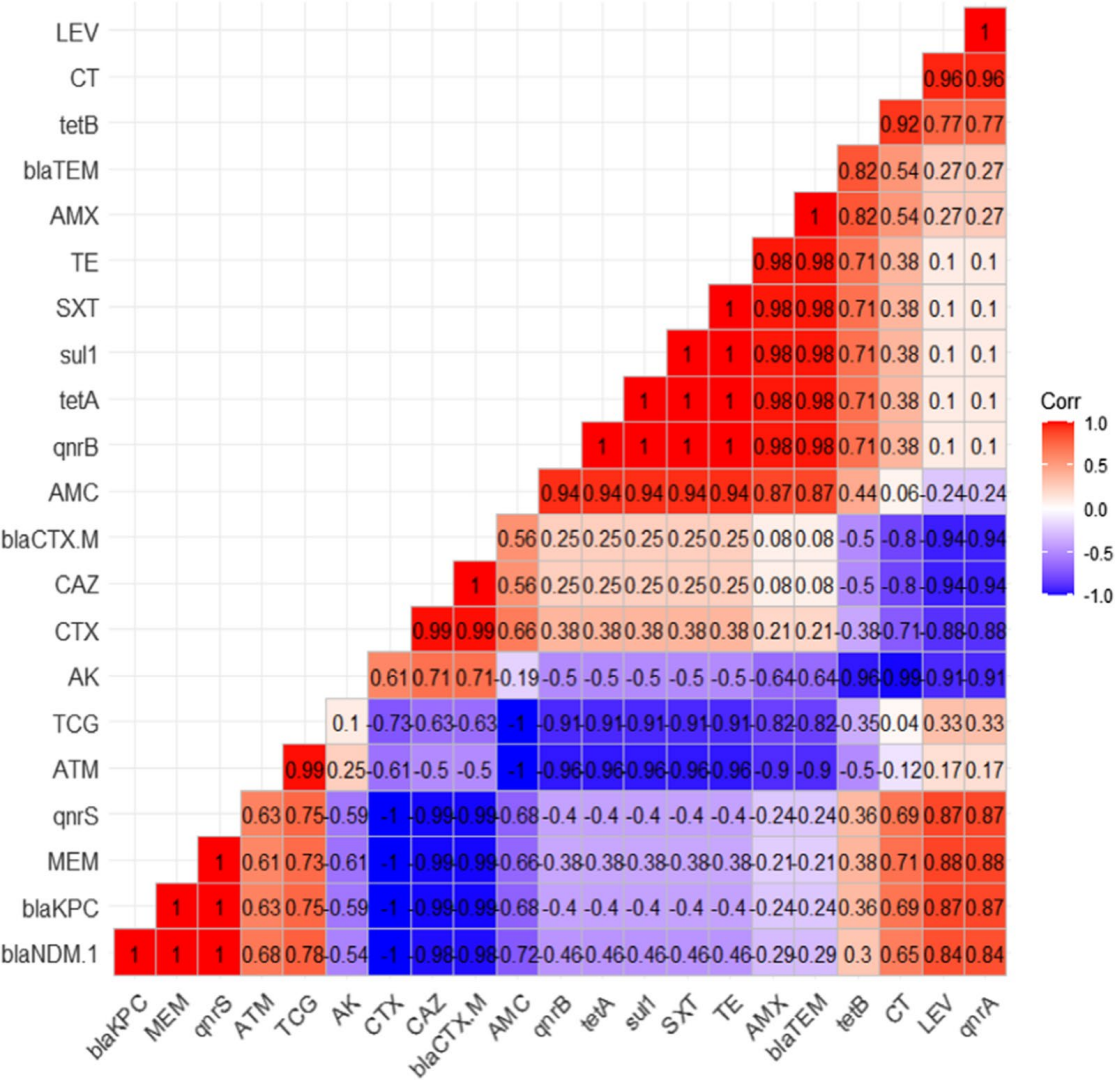
The heat map shows the correlation between the phenotypic antibiotic-resistant and corresponding genes

The correlation analysis among the antimicrobial agents and the corresponding resistance genes revealed significant positive correlations between: *sul*1gene and SXT (r = 1); *bla*_TEM_ gene and AMX (r = 1); *bla*_CTX-M_ and CAZ (r = 1); *tet*A gene and TE (r = 1); *qnr*A gene and LEV (r = 1); *bla*_KPC_ gene and MEM (r = 1); *bla*_NDM-1_ gene and MEM (r = 1); *bla*_TEM_ gene and AMC (r = 0.87); *bla*_CTX-M_ gene and CAZ (r = 0.87) *tet*B gene and TE (r = 0.71) as illustrated in Fig. 5.

## Discussion

The current study was directed to investigate the rate, antimicrobial resistance patterns, screening of virulence and antimicrobial resistance genes in STEC isolated from dogs suffering from hemorrhagic diarrhea. The clinically examined diseased dogs suffered from fever, vomiting, dehydration, and foul-smelling bloody diarrhea. Our findings agreed with those reported by (Nayel et al. 2019). Bloody diarrhea is attributed mainly to the destruction of the epithelium of the intestinal crypts and subsequent villous atrophy (Unterer and Busch 2020). Moreover, dehydration was also a characteristic clinical sequel caused by losing fluids due to vomiting and diarrhea (Sykes and Greene 2013).

In the present study, the phenotypic characteristics of the recovered *E.* *coli* isolates are similar to those reported by (Sengupta et al. 2011). Besides, the prevalence of *E.* *coli* is nearly similar to that reported by a previous study in urban dogs in France (24.5%) (Haenni et al. 2014). Moreover, a lower prevalence was reported in Tunisia (17.5%) (Sallem et al. 2013). Furthermore, a higher prevalence of *E.* *coli* was reported in Brazil (31.3%) (Carvalho et al. 2016). These disparities are attributed mainly to geographical variation, differences in the hygienic conditions, and the time of investigations conduction (Carvalho et al. 2021). Regarding *E.* *coli* serogrouping, the retrieved serovars are similar to those reported by Jay-Russell et al. (2014). The determination of *E.* *coli* serotypes reflects epidemiological and public health importance. All the examined *E.* *coli* serotypes (O111, O146, and O26) were positive for Congo-red binding that emphasized the virulence and invasiveness of the tested serovars. Furthermore, all the non-typeable isolates were negative. These results are consistent with those reported by (Algammal et al. 2020b).

In the present work, the obtained isolates displayed a remarkable resistance to trimethoprim-sulfamethoxazole, tetracycline, amoxicillin, amoxicillin-clavulanic acid, ceftazidime, and cefotaxime, which is greatly threatening public health. Our findings agreed with those recorded by Jay-Russell et al. (2014) and Gupta et al. (2017). Recently, antimicrobial resistance has increased globally due to the widespread unregulated application of antimicrobial agents in the health and veterinary sectors and the dissemination of antibiotic-resistant genes carried on the bacterial chromosome or plasmid (Yamamoto et al. 2013).

In the present study, the PCR revealed that the *stx*1 gene is the most predominant virulence gene associated with STEC isolated from diseased dogs. Our findings emphasized the presence of the *stx*1 gene in combination with the *eae*A and *hly*A genes and/or the *stx*2 gene. On the other hand, a previous study reported that the *stx*2 gene was the most predominant virulence gene (23%) in STEC strains isolated from diarrheic dogs in the USA (Staats et al. 2003). The pathogenicity of Shiga toxin-producing *E.coli* is attributed mainly to a variety of virulence determinants, including 1-Shiga toxins (encoded by *stx*1 and *stx*2 genes), which regularly destruct the eukaryotic ribosomes and prevent protein-biosynthesis in the host, 2-Intimin (encoded by the *eae* gene) that is responsible for *E.* *coli* attachment to the intestinal mucosa, and 3-Hemolysin-A (plasmid gene EHEC-*hly*A) is an exotoxin that lyses the erythrocytes in the intestinal mucosa (El-Baky et al. 2020). The virulence genes detected in *E.* *coli* of animal origin showed more toxin expression than those obtained from human strains (Keen and Elder 2002).

Regarding the multidrug resistance (MDR) profiles and the dissemination of antimicrobial resistance genes, our findings emphasized that most obtained isolates express MDR to 4 or more different antimicrobial classes and harbored *sul*1, *tet*A, *tet*B, *bla*_TEM_, *bla*_CTX.M-1_, and *qnr*A genes. The existence of MDR-*E.* *coli* reflects a public health alarm and suggests a poor prognosis of diseases induced by these isolates. Pet animals, especially dogs, are known as potential reservoirs of antimicrobial resistance. However, the available data are limited. The extensive application of antibiotics in both veterinary practice and the health sector predispose to the emergence of new superbugs. A recent study by Joosten et al. (2020) reported that 16% of the recovered *E.* *coli* isolates from dogs and cats from three European countries were recognized as multidrug-resistant strains (resistant to 6 antibiotics). One *E.* *coli* strain from Italy was resistant to 10 different antibiotics. Moreover, three *E.* *coli* strains (2 from Italy and one from Belgium) were multidrug-resistant to ampicillin, cefotaxime, and ceftazidime. The Extended-spectrum β-lactamases (ESBLs) are responsible for the hydrolysis of Broad-spectrum β-lactam antibiotics such as penicillins and cephalosporins. ESBLS are frequently detected in *E.* *coli* (Schultz et al. 2015). The high prevalence of the *bla*_TEM_ and *bla*_CTX-M_ genes among the retrieved *E.* *coli* strains enables them to resist penicillins and cephalosporins.

Tetracycline resistance is mainly attributed to tetracycline resistance genes. In the current study, the *tet*A gene was detected frequently in the recovered MDR *E.* *coli* strains, either alone or combined with the *tet*B gene. These findings agreed with a previous study performed on *E.* *coli* isolated from pigs in Spain (Jurado-Rabadán et al. 2014). Plasmid-mediated quinolone resistance (PMQR) was reported firstly in *K.* *pneumoniae* in the USA in 1998s. The *qnr*A gene codes for a 218 amino acid protein (QnrA protein) that prevents the binding of quinolones to DNA gyrase. Moreover, two other PMQR genes, including *qnr*B and *qnr*S genes, have been recognized to code for the same protein family and share 41% and 60% amino acid identity with QnrA, respectively (Robicsek et al. 2006). Alarmingly, in the present study, three *E.coli* strains were carbapenem-resistant. Worldwide, the existence of carbapenem-resistant strains is somewhat low; however, it has increased over time. The resistance to carbapenems is endorsed by *bla*_KPC_ and *bla*_NDM-1_ resistance genes (Cole et al. 2020). Moreover, a lower resistance rate against amikacin was recorded in the current study. These findings agreed with those reported by Qekwana et al. (2018), who stated that the aminoglycoside resistance in *E.* *coli* strains isolated from urinary tract infections in dogs was relatively low (22%). This lower resistance rate could be attributed to the less frequent use of amikacin in treating bacterial infections in dogs in Egypt. The resistance mechanisms in *E.* *coli* include: a-A common resistance mechanism that occurs in antibiotics of the same class due to the expression of extended-spectrum β-lactamases or mutations in the penicillin-binding proteins, b-The repeated combined antimicrobial therapies, and c-The association between the antimicrobial resistance genes; this type exhibits a vital role in the occurrence of resistance to various antimicrobial agents (Algammal et al. 2020a, 2020b).

As far as the authors know, this is the first study that reported the existence of MDR-Shiga toxigenic *E.* *coli* (STEC) in dogs in Egypt. The *stx*1 gene is the most predominant Shiga toxin gene, found mainly in combination with the *eae*A and *hly*A genes and/or the *stx*2 gene. The emerging MDR-STEC in dogs commonly harbors *bla*_TEM_, *bla*_CTX-M_, *sul*1, *tet*A, *tet*B, and *qnr*A resistance genes. Meropenem, levofloxacin, and tigecycline exhibited talented antimicrobial activity against MDR-STEC isolated from dogs. The regular conduction of antimicrobial susceptibility testing is necessary to detect the most effective antibiotic and determine the emergence of new MDR strains. The emergence of virulent MDR-STEC strains comprises a vital threat to pet animals and human health, especially carbapenem-resistant strains harboring *bla*_KPC_ or *bla*_NDM-1_ resistance genes.

## Data Availability

Not applicable.
